# MafA is a Key Molecule in Glucose and Energy Balance in the Central Nervous System and Peripheral Organs

**Published:** 2011-03

**Authors:** Mariko Tsuchiya, Ken Tsuchiya, Kazuki Yasuda, Mikiko Fujita, Akira Takinishi, Maiko Furukawa, Kosaku Nitta, Atsushi Maeda

**Affiliations:** 1 *Institute of Geriatrics, Tokyo Women’s Medical University, Japan;*; 2 *Department of Medicine IV, Tokyo Women’s Medical University, Japan;*; 3 *International Medical Center of Japan, Tokyo, Japan*

**Keywords:** central nervous system, MafA, microarray, siRNA, transcriptional factor

## Abstract

MafA is a strong transactivator of insulin in pancreatic β cells. Elucidating the profile of MafA action in organs other than the pancreas is essential. We established an mRNA interference technique that modifies the level of target mRNAs in mice *in vivo*. After rapidly injecting MafA-siRNA, the resulting changes in the gene profile were analyzed using a microarray system. Significant suppression of the MafA mRNA levels was observed in the pancreas, liver, adipose tissue, and brain of siRNA-injected mice. As we reported previously, the down-regulation of insulin mRNA and adipocytokines was observed in the pancreas, and MafA siRNA caused alterations in the expressions of genes related to lipid metabolism and cell growth in the liver, and the attenuation of cell differentiation in cultured adipocytes. In addition to the effects on these organs, MafA expression was immunohistochemically detected in the brain in our preliminary data, and the expression level in siRNA-treated mice was significantly suppressed. The expressions of the affected genes were distinct, including growth hormone, vasopressin, hypocretin, and pro-melanin-concentrating hormone, were almost completely down-regulated (to ~1/100). These results suggested that MafA is likely involved in the regulation of hormonal systems related to glucose metabolism, and MafA is likely positioned near the beginning of the cascade or may influence the expressions of the above-mentioned genes in coordination with other factors in brain tissue. Taken together, the findings in this study suggested that MafA functions as a transcription factor with distinct activities in each organ and is cross-linked in several organs.

## INTRODUCTION

The large Maf proteins are a family of transcription factors characterized by a typical bZip structure, which is a motif for protein dimerization and DNA binding. These proteins reportedly regulate several distinct developmental processes, cell differentiation, and the establishment of cell functions. One of the large Mafs, MafA protein, has been established as a strong transactivator of insulin in pancreatic β cells (1-4), and MafA reportedly regulates developmental processes in the pancreas, cell differentiation, and the establishment of endocrine and non-endocrine cell function in coordination with other kinds of Mafs (5, 6). On the other hand, Mafs are well known to play important roles in a variety of developmental and differentiation processes in many organs, tissues (7), and cells, including the pancreas (8), lens (9), myeloma cells (10), and cartilage (11).

As the role of MafA is not limited to pancreatic β cells, we speculated that MafA might be a key molecule in the networking of glucose and lipid metabolism (12). On the other hand, glucose is essential for energy metabolism in brain tissue; thus, MafA may play special roles in the central nervous system (13, 14). Since little is known regarding the actions of MafA in the brain, the aim of this study was to elucidate the role of MafA in the central nervous system, in which glucose is the only source of energy.

## MATERIAL AND METHODS

### *In vivo* suppression of MafA mRNA using siRNA: Intravenous hydrodynamic method

**Animal preparation.** Male mice between the ages of six to eight weeks were maintained under stable conditions. All the animal procedures were performed in accordance with the guidelines set by the National Institute of Health and the Institutional Animal Care and Use Committee of Tokyo Women’s Medical University.

### MafA siRNA and SiRNA injection

A designed siRNA oligomer constructed in a plasmid was purchased from Takara Bio (Takara Bio Co., Japan). The target sequence and designed siRNA sequence are shown in Table 1.

**Table 1:** Target sequence and designed siRNA sequence

MafA NM_194350		

Target sequence	AGCGGGACCCTGTACAAGGA	
Sense oligo	gtttAGTGGGACTTGTACAGGGAACGTGTGCTGTCCGTTCCTTGTACAGGTCCCGCTTTTTT	
Antisense oligo	atgcAAAAAAGCGGGACCTGTACCAGGAACGGACAGCACACGTTCCCTGTACAAGTCCCACT	
Vector	pcPURmU6icassette	
Stop siRNA		
Sense Oligo	GTTTTTTTTTT	
Antisense Oligo	ATGCAAAAAAA	
Primers for real-time PCR		
MafA	Forward: CCAGCTGGTATCCATGTCC	Reverse: TTCTGTTTCAGTCGGATGACC

Anesthetized mice were intravenously injected with the siRNA contained in the plasmid using the hydrodynamic method according to the procedure described by Hamar (15). Briefly, immediately after immersing the tail in a 55°C warm water bath for 5 seconds to dilate the tail veins, siRNA dissolved in TransIT^R^-QR Hydrodynamic Delivery Solution (Mirus Bio Corporation, Madison, WI) according to the manufacturer’s instructions was rapidly injected into the tail vein within 5 seconds.

### DNA microarray analysis

The DNA microarray analysis was performed as described previously (16). Briefly, Affymetrix Gene Chip technology was used as follows. cDNA was synthesized from the total RNA using a Gene Chip Expression 3’-Amplification Reagents One-Cycle cDNA Synthesis Kit (Affymetrix, Santa Clara, CA). The total RNA (8 μg) was annealed to T7-Oligo (dT) Primer (50 μM) at 70°C for 10 minutes, and reverse transcription was carried out. The reaction mixture was incubated at 16°C for 2 hours, 2 μL of T4 DNA polymerase at 5 U/μL was added, and incubation at 16°C was continued for 5 minutes. After termination, biotin-labeled cRNA was synthesized using Gene Chip Expression 3’-Amplification Reagents for IVT Labeling (Affymetrix). The reaction was allowed to proceed at 37°C for 16 hours in a mixture containing template cDNA, RNAase-free water, 10 x IVT Labeling Buffer, IVT Labeling NTP Mix, and IVT Labeling Enzyme. A 15 μg sample of the fragmented cRNA was hybridized to the GeneChip Mouse Genome 430 2.0 Array Set (Affymetrix) at 45°C in a rotisserie hybridization oven at 60 rpm for 16 hours. The probe arrays were exposed to antibody solution (1 x MES solution, 0.005% antiform, 2 mg/mL acetylated BSA, 0.1 μg/μL normal goat IgG [Sigma, St. Louis, MO], 3 μg/μL goat-anti-streptavidin, and biotinylated antibody [Vector Laboratories, Burlingame, CA]) at 35°C for 5 minutes; after washing and staining, the probe array was scanned twice at a 3 μm resolution using a GeneChip System confocal scanner (Hewlett-Packard, Santa Clara, CA) controlled by GeneChip 3.1 software (Affymetrix).

### Reverse-transcription (RT) and real-time PCR

RNA isolation and real-time PCR were performed as previously described (17). Briefly, total RNA was isolated from the pancreas, liver, adipose tissue and brain using the RNeasy Plus Mini Kit (QIAGEN). Relative quantitation using the real-time PCR method was performed using SYBR Green PCR Reagents and an ABI PRISM 7700 Sequence Detection System (PE Applied Biosystems, Foster City, CA) according to the manufacturer’s instructions. Reactions were performed using 1.0 μL of RNA at a concentration of 40 ng/μL in a reaction volume of 25 μL. RT was performed at 37°C for 120 minutes, followed by PCR consisting of AmpliTaq activation for 10 minutes at 95°C, then 40 cycles of heating to 95°C for 15 seconds and cooling to 60°C for 1 minute. The mRNA levels were normalized to the levels of GAPDH mRNA. Specific primers for the real-time PCR were designed and are summarized in Table 2.

**Table 2:** Down-regulated and up-regulated genes and their primers for real-time PCR

ACCESSION No.	DEFINITION	F	R

Down-regulation			
NM_029971	Mus musculus pro-melanin-concentrating hormone (Pmch), mRNA	gccccttctctggaacaata	ttggagcctgtgttctttga
NM_009732	Mus musculus arginine vasopressin (Avp), mRNA	ccaggatgctcaacactacg	ctcttgggcagttctggaag
NM_010410	Mus musculus hypocretin (Hcrt), mRNA	ttggaccactgcactgaaga	cccagggaacctttgtagaag
BC061215	Mus musculus pro-opiomelanocortin-alpha, mRNA (cDNA clone MGC:74362 IMAGE:30253829), complete cds	gtccctccaatcttgtttgc	cctgagcgactgtagcagaa
NM_008117	Mus musculus growth hormone (Gh), mRNA	catggaattgcttcgcttct	caggctgttggtgaaaatcc
Up-regulation			
BC024702	Mus musculus transthyretin, mRNA (cDNA clone MGC: 18651 IMAGE:4192268), complete cds.	ggacaccaaatcgtactggaa	agtcgttggctgtgaaaacc
NM_021301	Mus musculus solute carrier family 15 (H+/peptide transporter), member 2 (Slc15a2), mRNA	gacattccaaagcgacaaca	atcctggtcagtgccttcac
NM_010234	Mus musculus FBJ osteosarcoma oncogene (Fos), mRNA	tgccaatctgctgaaagaga	atctcctctgggaagccaag
BC024515	Mus musculus gastrin releasing peptide, mRNA (cDNA clone MGC:37475 IMAGE:4984025) complete cds	caagggatttgctggacct	cccaagtaggctggagactg

### Western blotting

Frozen organs were immersed in a lysis buffer (20 mM Tris buffer, pH7.5, containing 1 mM phenylmethylsulfonyl fluoride and 10 μg/mL aprotinin from bovine lung [Wako, Tokyo], 2 mM DL-dithiothreitol, 1% polyoxyethylene sorbitan monolaurate, and 1 mM ethylenediamine tetraacetate) and then homogenized on ice. After centrifugation at 5000 r.p.m. for 10 minutes, 20 μg of protein from each sample was suspended in a loading buffer, separated on a 10% polyacrylamide gel (Readygels J, BIO-RAD, Tokyo, Japan), and electrophoretically transferred to a nitrocellulose membrane. The membranes were blocked with 5% skim milk for 1 hour at room temperature. A primary antibody against MafA (BL1069; Bethyl Laboratories Inc.) was used at a dilution of 1:600 and was applied overnight at 4°C. After two 10-minute washing steps with washing buffer (0.3% Tween20 in PBS), the membrane was incubated with horseradish-peroxidase-conjugated immunoglobulins (DAKO, Tokyo, Japan) for 1 hour at room temperature, then with an ECL western blotting system (Amersham Biosciences, Tokyo). Finally, LUMINESCENT IMAGE ANALYZER LAS-100plus (FUJI FILM) was exposed to the membrane.

### Statistical analysis

All the results are expressed as the means ± S.E.M. Differences in the expression levels were analyzed using a paired Student t-test with Bonferroni correction, and differences were considered significant when the p value was <0.05. The experiments were repeated 5 times.

## RESULTS

### siRNA-induced suppression of MafA in mice

**Figure 1:**
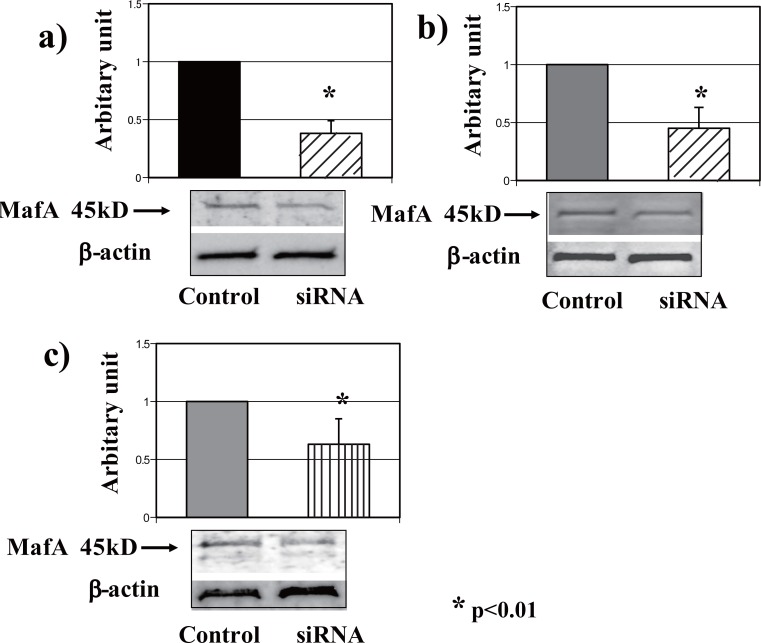
Suppression of MafA in mice using siRNA. SiRNA significantly suppressed MafA mRNA; representative western blots are shown for the a) pancreas, b) liver, and c) adipose tissue.

The MafA mRNA level was significantly suppressed in the pancreas, liver and adipose tissue of siRNA-injected mice. An almost 60% reduction in MafA expression in the pancreas was achieved *in vivo* using the intravenous hydrodynamic method of administering siRNA. The suppressive effect of siRNA was assessed by comparing the results with those in the pancreases of mice injected with control siRNA. The relative mRNA expression levels were determined using the real-time PCR method to compare the samples of siRNA and control siRNA. The down-regulation of MafA mRNA in the liver (55%) and adipose tissue (35%) was also observed using siRNA (Figure 1).

To confirm the expression and the altered expression levels of MafA in each organ and tissue, western blotting was performed using an MafA-specific antibody. As shown in representative blots in Figure 1, the changes in the protein expression levels paralleled the changes in the mRNA levels.

### MafA expression in the brain

**Figure 2:**
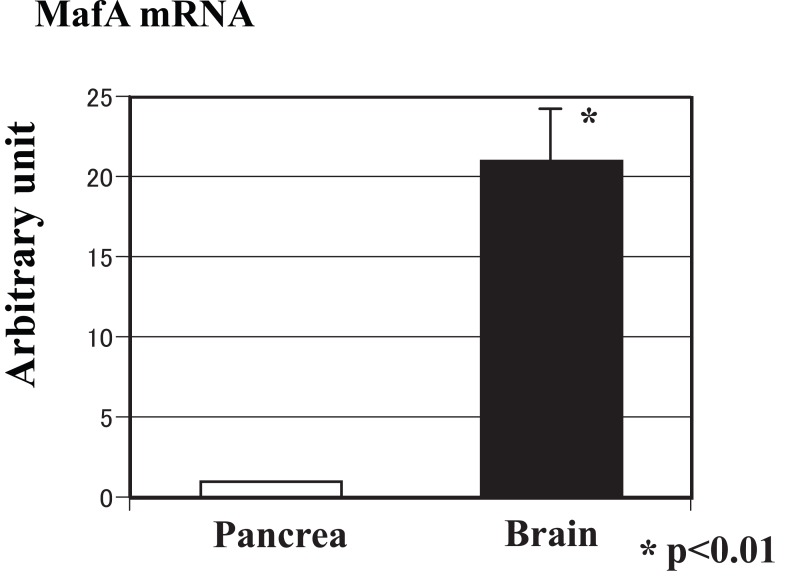
Expression level of MafA mRNA in the brain. The expression level of MafA mRNA in the brain was 20 times that of MafA mRNA in the pancreas, as assessed using real-time PCR.

In addition to the expression levels in peripheral organs, MafA expression was also examined in the central nervous system. As shown in Figure 2, the expression level of MafA mRNA in the brain was 20 times that in the pancreas, as assessed using real-time PCR.

**Figure 3:**
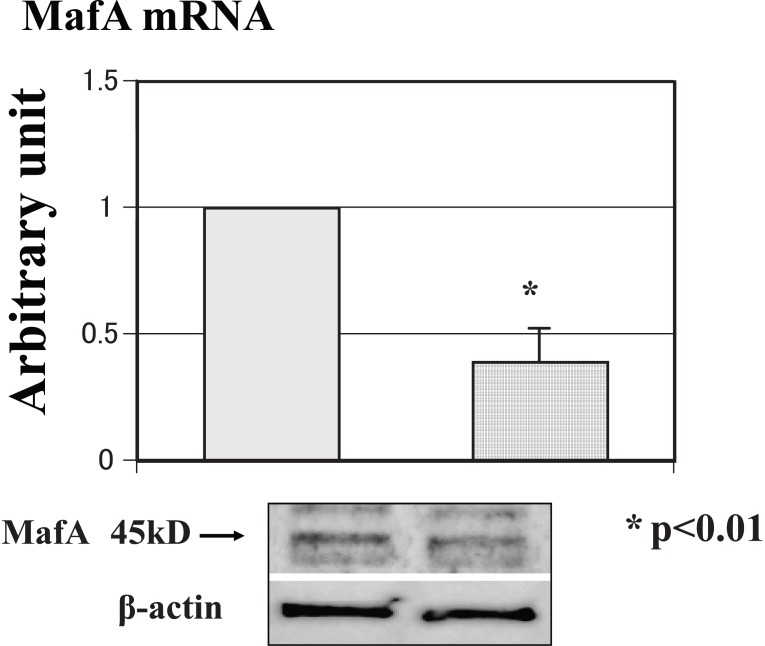
Expression level of mRNA and a representative blot in the brain tissue. The mRNA and protein expression levels of MafA were significantly down-regulated by the siRNA.

The expression level of MafA mRNA in siRNA-treated mice was approximately 60% lower than that of mRNA in stop-siRNA-treated mice. A representative western blot analysis was performed to confirm the real-time PCR results. Figure 3 shows the mRNA expression level and a representative blot, demonstrating that the mRNA and protein expressions of MafA were both significantly down-regulated by the siRNA.

### Changes in the gene profile of the brain after treatment with MafA siRNA, as determined using a microarray analysis

The gene profile of siRNA-treated mice was analyzed at 24 hours after injection using the hydrodynamic method. The MafA and insulin mRNA levels in the pancreases of these mice were suppressed by 30%, compared with the stop-siRNA-treated mice.

**Figure 4:**
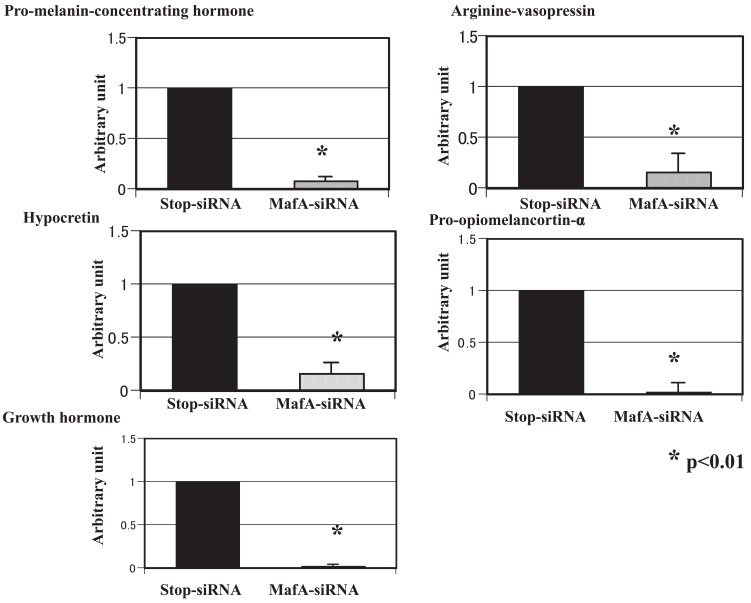
Genes with down-regulated expression levels in the brain tissue. Real-time PCR with specific primers was performed, and the relative changes in the expression levels of the down-regulated genes are shown.

**Figure 5:**
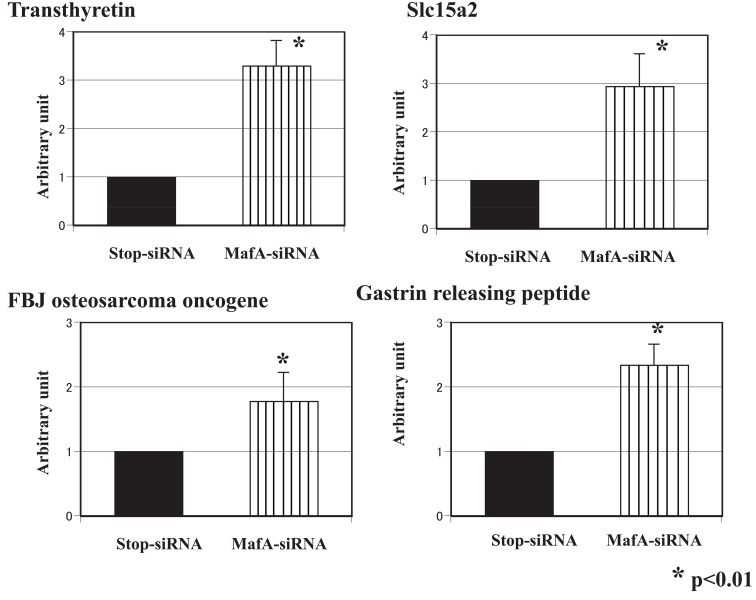
Genes with up-regulated expression levels in the brain tissue. Real-time PCR with specific primers was performed, and the relative changes in the expression levels of the up-regulated genes are shown.

The gene expression profiles of the brain in MafA-siRNA-treated mice were analyzed using the microarray method and compared with those of control-siRNA-treated mice. The expression levels of several genes were altered: 20 genes were up-regulated, and 15 genes were down-regulated. Genes with an expression level altered by more than 50% were selected for a detailed analysis. We performed real-time PCR with specific primers to confirm and observe the relative changes in the expression levels of these genes (listed in Table 2). Pro-melanin-concentrating hormone, arginine vasopressin, hypocretin, pro- opiomelanocortin- alpha and growth hormone were down-regulated (Figure 4), while transthyretin, solute carrier family 15, FBJ osteosarcoma oncogene and gastrin-releasing peptide were up-regulated (Figure 5). The affected gene expression profiles were distinct from those in other organs and tissues, and several genes were almost completely down-regulated in the brain tissue.

## DISCUSSION

This study examined MafA-related gene expression in vivo in mouse organs using RNA interference and the hydrodynamic method. We used in vivo siRNA injection and observed a successful targeted gene suppression in the pancreas, liver, adipose tissue and brain. We used a siRNA-expressing plasmid DNA (pDNA) instead of the oligonucleotide form of siRNA with the aim of achieving a longer lasting suppression of the mRNA (18). The degree of suppression was dependent on the dose of siRNA-expressing pDNA, and the reduction in transgene expression became apparent 1 day after the injection.

In our previous study, we showed that the suppression of MafA mRNA expression in the pancreas *in vivo* induced the down-regulation of gene expression of pancreatic hormones as well as adipocytokines (adipsin and adiponectin) (19). MafA is closely related to pancreatic β cell differentiation and normal function, and several recent reports have revealed a role of MafA in the final differentiation or replication of β cells (12, 20). In addition to its effects on β cells, MafA may be involved in adipocyte differentiation and the regulation of lipid metabolism through the adipocytokine network in the pancreas. We have shown in a previous study that MafA interference induced the down-regulation of adiponectin and adipsin (19). Adiponectin has recently been reported to exert insulin-sensitizing, anti-atherogenic, and anti-inflammatory actions but does not affect insulin secretory function (21). Although the precise mechanism and cross-linking behavior were not clarified in this study, Maf-regulated pancreatic endocrine function appears to affect the adipocytokine network (22, 23). In the liver, MafA mRNA suppression revealed that the expressions of genes related to lipid metabolism or cell growth were altered (data not shown). Taken together, these findings suggest that mechanisms allowing cross talk with distant organs may exist, coordinating a variety of biological processes including energy metabolism, the inflammatory cascade, and insulin-stimulated secretion.

Thus, we focused on the changes in the gene profile in the central nervous system, since the MafA mRNA level was simultaneously and significantly down-regulated in the brain tissue using our MafA siRNA technique. The results of the affected gene expressions in the brain tissue were distinct, comparing with those for other organs and tissues, in that several genes were almost completely down-regulated (Figure 4). These genes are related to food consumption or metabolism. For example, growth hormone is a well-known hormone regulating growth and development that has an anti-insulinergic activity. Arginine vasopressin regulates urine osmolarity as well as social behavior, pairing, brain edema, and low anxiety-related behavior. Moreover, pro-melanin-concentrating hormone (MCH) is involved in body weight regulation (24). The hypothalamic expression of MHC mRNA is upregulated during starvation in mice. MHC overexpression leads to obesity and an increased susceptibility to high-fat feeding, while the ablation of MCH has been reported to promote fat loss mainly by increasing energy expenditure (25, 26). Orexin exerts essential functions as a regulator of behavioral arousal, sleep and wakefulness (27). Orexin may have a role in the brain-gut network of orexin-containing cells that appear to play a role in the acute regulation of energy homeostasis (28). Pro-opiomelanocortin-alpha (alpha-melanocyte stimulating hormone) is a tridecapeptide cleaved from pro-opiomelanocortin that acts to inhibit food intake.

On the other hand, the up-regulation of four genes was confirmed using RT-PCR, although the magnitude of the changes was not as prominent as that for the down-regulated genes. Transthyretin (TTR), which inhibits the aggregation of amyloid-beta, is a serum and cerebrospinal fluid carrier of the thyroid hormone thyroxine (T4) and retinol (29). Slc15a2 acts as a high-affinity proton-dependent peptide transporter that may transport peptides from the cerebrospinal fluid to the blood (30). FBJ osteosarcoma oncogene is an immediate early gene encoding a nuclear protein involved in signal transduction. Gastrin-releasing peptide, also known as GRP, is an important regulatory molecule that has been implicated in a number of physiological and pathophysiological processes in humans. These smaller peptides regulate numerous functions of the gastrointestinal and central nervous systems, including the release of gastrointestinal hormones, smooth muscle cell contraction, and epithelial cell proliferation. The precise relationship or significance of MafA linking all these genes cannot be discussed here, but the characteristics of all these genes are closely related to nutrition, energy balance and feeding behavior.

These results suggested that MafA is likely involved in the regulation of hormonal systems related to glucose metabolism, in which regulation by MafA likely occurs near the beginning of the cascade or acts directly on the expression of these genes in coordination with other factors in the brain tissue. On the other hand, glucose is essential for energy metabolism in brain tissue; thus, Maf is likely involved in the formation of a network or the mediation of cross-talk among multiple organs, including the central nervous system, with regard to glucose, lipid, and energy balance. Furthermore, MafA acts as a switch for the expression of related genes; consequently, the expression of each gene and its intensity during the time course should be monitored. The Maf family, especially MafA, plays diverse roles as transcriptional factors in the establishment of energy balance in peripheral organs, such as the pancreas, liver, and adipose tissue. Finally, MafA is likely to play potential roles in immune reactions, inflammation, development, and regeneration, triggering rapid changes in direction mediated by alterations in gene profiling.

## References

[1] Olbrot M, Rud J, Moss LG, Sharma A (2002). Identification of β-cell-specific insulin gene transcription factor RIPE3b as mammalian MafA. Proc. Natl. Acad. Sci. USA.

[2] Kataoka K, Han S, Shioda S (2002). MafA is a glucose-regulated and pancreatic β-cell-specific transcriptional activator for the insulin gene. J. Biol. Chem.

[3] Matsuoka T, Artner I, Henderson E (2004). The mafA transcription factor appears to be responsible for tissue-specific expression of insulin. Proc. Natl. Acad. Sci. USA.

[4] Zhao L, Guo M, Matsuoka T (2005). The islet β cell-enriched mafA activator is a key regulator of insulin gene transcription. J. Biol. Chem.

[5] Matsuoka T, Zhao L, Artner I (2003). Members of the large maf transcription family regulate insulin gene transcription in islet β cells. Mol. Cell Biol.

[6] Kataoka K, Shioda S, Ando K (2004). Differentially expressed maf family transcription factors, c-maf and mafA, activate glucagons and insulin gene expression in pancreatic α-and β-cells. J. Mol. Endocrinol.

[7] Kataoka K, Nishizawa M, Kawai S (1993). Structure-function analysis of the maf oncogene product, a member of the b-Zip protein family. J. Virol.

[8] Tsuchiya M, Taniguchi S, Yasuda K (2006). Potential roles of large mafs in cell lineages and developing pancreas. Pancreas.

[9] Sakai M, Imaki J, Yoshida K (1997). Rat maf related genes: specific expression in chondrocytes, lens and spinal cord. Oncogene.

[10] Chesi M, Bergsagel PL, Shonukan OO (1998). Frequent dysregulation of the c-maf proto-oncogene at 16q23 by translocation to an Ig locus in multiple myeloma. Blood.

[11] MacLean HE, Kim JI, Glimcher MJ (2003). Absence of transcription factor c-maf causes abnormal terminal differentiation of hypertrophic chondrocytes during endochondral bone development. Dev. Biol.

[12] Zhang C, Moriguchi M, Kajihara M (2005). MafA is a key regulator of glucose-stimulated insulin secretion. Mol. Cell Biol.

[13] Yamada T, Oka Y, Katagiri H (2008). Inter-organ metabolic communication involved in energy homeostasis: potential therapeutic targets for obesity and metabolic syndrome. Pharmacol Ther.

[14] Yamada T, Katagiri H (2007). Avenues of communication between the brain and tissues/organs involved in energy homeostasis. Endocr. J.

[15] Hamar P, Song E, Kokeny G (2004). Small interfering RNA targeting Fas protects mice against renal ischemia-reperfusion injury. Proc. Natl. Acad. Sci. USA.

[16] Shirota S, Yoshida T, Sakai M (2006). Correlation between the expression level of c-maf and glutathione peroxidase-3 in c-maf -/- mice kidney and c-maf overexpressed renal tubular cells. Biochem. Biophys. Res Commun.

[17] Yoshida T, Tang SS, Hsiano LL (2002). Global analysis of gene expression in renal ischemia-reperfusion in the mouse. Biochem. Biophys. Res Commun.

[18] Kobayashi N, Matsui Y, Kawase A (2004). Vector-based *in vivo* RNA interference: dose- and time-dependent suppression of transgene expression. J. Pharmacol Exp. Ther.

[19] Tsuchiya M, Yoshida T, Taniguchi S (2007). *In vivo* suppression of mafA mRNA with siRNA and analysis of the resulting alteration of the gene expression profile in mouse pancreas by the microarray method. Biochem. Biophys. Res Commun.

[20] Harmon JS, Stein R, Robertoson RP (2005). Oxidative stress-mediated, post-translational loss of mafA protein as a contributing mechanism to loss of insulin gene expression in glucotoxic beta cells. J. Biol. Chem.

[21] Staiger K, Stefan N, Staiger H (2005). Adiponectin is functionally active in human islets but not affect insulin secretory function or β-cell lipoapoptosis. J. Clin. Endocrinol Metab.

[22] Poitout V, Hagman D, Stein R (2006). Regulation of the insulin gene by glucose and fatty acids. J. Nutr.

[23] Okamoto Y, Kihara S, Funahashi T (2006). Adiponectin: a key adipocytokine in metabolic syndrome. Clin. Sci.

[24] Ludwig DS, Tritos NA, Mastaitis JW (2001). Melanin-concentrating hormone overexpression in transgenic mice leads to obesity and insulin resistance. J. Clin. Invest.

[25] Qu D, Ludwig DS, Gammeltoft S (1996). A role for melanin-concentrating hormone in the central regulation of feed behaviour. Nature.

[26] Shimada M, Tritos NA, Lowell BB (1998). Mice lacking melanin-concentrating hormone are hypophagic and lean. Nature.

[27] Sakurai T, Amemia A, Ishii M (1998). Orexins and Orexin Receptors: A Family of Hypothalamic Neuropeptides and G Protein-Coupled Receptors that Regulate Feeding Behavior. Cell.

[28] Kirchgessner AL (2002). Orexins in the Brain-Gut Axis. Endocr. Rev.

[29] Ingenbleek Y, Young V (1994). Transthyretin (Prealbumin) in health and disease: Nutritional implications. Annu. Rev. Nutr.

[30] Shu C, Shen H, Teuscher NS (2002). Role of PEPT2 in peptide/mimetic trafficking at the blood-cerebrospinal fluid barrier: studies in rat choroids plexus epithelial cells in primary culture. J. Pharmacol Exp. Ther.

